# Association between live microbe intake and severe headache or migraine: evidence from NHANES 1999–2004

**DOI:** 10.3389/fnut.2025.1547371

**Published:** 2025-05-06

**Authors:** Rongjiang Xu, Xiangmin Yu, Ruonan Zhang, Xiaonuo Xu, Xiaoping Fan, Liang Dong, Jiying Zhou

**Affiliations:** ^1^Department of Neurology, The First Affiliated Hospital of Chongqing Medical University, Chongqing, China; ^2^Phase I Clinical Research Center, The First Affiliated Hospital of Chongqing Medical University, Chongqing, China

**Keywords:** dietary live microbe, severe headache or migraine, microbiological therapy, NHANES, cross-sectional study

## Abstract

**Objective:**

The pathogenesis of migraine is not fully understood until now. This study was designed to explore whether the intake of live dietary microbes could be used as an auxiliary means for the treatment of severe headache and migraine.

**Methods:**

Data used in this study were came from the US National Health and Nutrition Examination Survey (NHANES) from 1999 to 2004. Participants were divided into three groups according to the dietary live microbe classification system, namely low, medium and high dietary live microbe groups. Weighted logistic regression models were used for statistical analysis.

**Results:**

A total of 13,443 participants were included in the present study. Compared with the low dietary live microorganism group, the migraine OR (95% CI) of medium-high dietary live microorganism group is 0.71 (0.63–0.81) and 0.73 (0.62–0.86), respectively, in the unadjusted model. After fully adjusting for confounding factors, patients in medium-high dietary live microbe group had a lower prevalence of migraine in contrast to those in low dietary live microbe group (Medium OR: 0.79, 95% CI: 0.68–0.93, *P* = 0.005; High OR: 0.82, 95% CI: 0.67–0.99, *P* = 0.047).

**Conclusion:**

Our study shows that a moderate-high intake of live dietary microbes is inversely associated with the prevalence of severe headache or migraine.

## 1 Introduction

Migraine is characterized by recurrent episodes of severe unilateral pulsating headaches accompanied by nausea, vomiting, and increased sensitivity to light and sound (1). Migraine is a serious and disabling neurovascular disorder that imposes a huge burden on people's lives and society (2). The results of the 2019 Global Burden of Disease Study show that migraine is the second leading cause of disability worldwide (3). About 1 billion people worldwide suffer from migraine, and in the United States, about 15% of adults experience a migraine attack each year (4). Currently, migraine is mainly treated with medication. However, due to poor long-term medication adherence and adverse drug reactions, in recent years, more and more researchers begun to focus on the important role of diet in migraine prevention and acute treatment (5).

Food-safe microbes obtained through daily diet may interact with the mucosal surface of the digestive tract to modulate the immune system, enhance intestinal function, and reduce the body's susceptibility to chronic diseases (6). Previous studies have shown that supplementation with probiotics or synthetics could significantly reduce the frequency of migraine attacks and the intensity of migraine. For example, a double-blind randomized controlled trial found that after female migraine patients received synbiotics intervention, the average monthly frequency of attacks decreased by 1.02, the use of analgesics decreased by 7.5%, and the levels of intestinal permeability markers decreased (7). Another meta-analysis showed that probiotics decreased the frequency (mean difference = −1.16), severity (mean difference = −1.07), and the monthly migraine days (mean difference = −3.02) (8). Furthermore, in patients with migraine combined with irritable bowel syndrome (IBS), a diet that eliminates IgG and combines with probiotics treatment not only improved headaches but also increased serotonin levels and reduced dependence on over-the-counter analgesics (9). Recently, Marco et al. (10) used the NHANES public database to propose a classification system for defining and estimating dietary intake of live microorganisms. Based on this classification system, several papers have explored the correlation between dietary live microorganisms and a variety of common clinical diseases (11–14).

However, no studies have investigated the relationship between dietary consumption of live microbes and migraine. Therefore, our study aimed to examine the association between dietary intake of live microbes and migraine using data from the NHANES survey conducted between 1999 and 2004.

## 2 Methods

### 2.1 Participants from NHANES

The data used in our study were derived from the National Health and Nutrition Examination Survey (NHANES). NHANES is a comprehensive data collection program hosted by the National Center for Health Statistics of the CDC to collect information on the health and nutritional status of the US population. It uses a stratified multi-stage probabilistic design to screen participants, including over-sampling of specific age and ethnic groups. The method allows for sample-weighted inference and provides a representative representation of the US population. Individuals provided written informed consent before participating in NHANES, while the publicly available NHANES database maintains anonymity by omitting patient identifiers.

**Figure 1:**
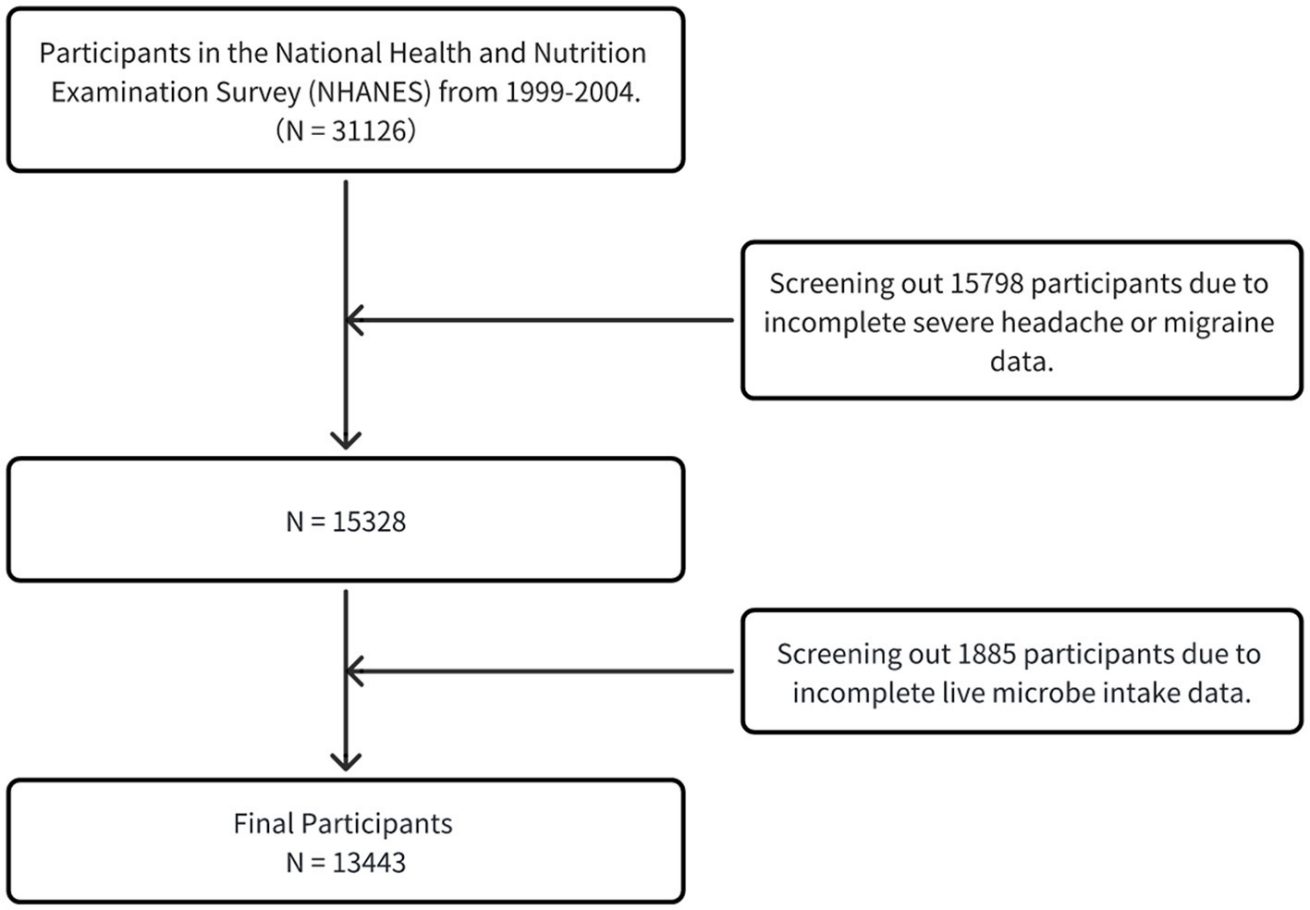
A flowchart showing the selection of study participants.

This analysis initially encompassed 31,126 adult participants aged 20 years and above. Subsequently, individuals without severe headaches or migraine (*n* = 15,798) were excluded, leaving 15,328 samples for subsequent refined analysis. On this foundation, participants in the Live Microbe Intake project (*n* = 1,885) were further excluded. Ultimately, a total of 13,443 participants constituted the base group for this study (Figure 1).

### 2.2 Ascertainment of severe headache or migraine

The assessment of severe headache or migraine in the NHANES database was primarily based on self-reported methods. Specifically, migraine status was determined through a “Miscellaneous Pain” section questionnaire administered during family interviews, which contained questions: “Have you had a severe headache or migraine in the past three months?” Participants who answered “yes” were classified as having migraine, while those who answered “no” were included in the control group. According to the American Migraine Epidemiology and Prevention Study, approximately 94% of individuals who self-reported severe headache were diagnosed with migraine or possible migraine according to the criteria of the International Classification of Headache Disorders, Second Edition (ICHD-2) (15). Therefore, in this study, participants who answered “yes” were considered to have migraines, and those who answered “no” were considered to have no migraine. This approach has precedent in previous studies, and people with severe headaches were more likely to have migraines, consistent with the findings of the American Migraine Epidemiology and Prevention Study (16, 17).

### 2.3 Live microbe intake

The researchers used a combination of face-to-face interviews and a 24-h dietary review to collect data on all foods and beverages consumed by the participants. Due to the participants' reliance on memory, this method may produce recall bias, But the National Health and Nutrition Examination Survey (NHANES) uses strict quality control procedures to reduce this bias. Interviewers use partial-size models, detailed question inquiries, and multiple repeated inquiries to help participants accurately recall dietary intake and reduce overreporting or underreporting. In addition, NHANES used automated systems to reduce interviewer bias and standardized data collection. The NHANES database covers 9,388 food codes, broken down into 48 subgroups. Four experts estimated the live bacteria content (per gram) in these foods (10). Foods were classified into three grades based on live bacteria content: low (<10^4^ CFU/g), medium (10^4^-10^7^ CFU/g), and high (>10^7^ CFU/g) (10). In the study, participants were divided into three groups based on diet live bacteria content: low diet live bacteria group (all foods were low), medium diet live bacteria group (medium content but no high content food), and high diet live bacteria group (containing high content food) (18). These assessments are based on reported values in professional literature, authoritative reviews, and extrapolations from the known effects of food processing on microbial viability, and are addressed through external consultation and discussion to resolve cases of inconsistent or conflicting data. Generally, foods that have been pasteurized or processed at high temperatures are typically categorized as low levels. Unpeeled fresh vegetables and fruits fall into the medium category, while unpasteurized fermented foods and probiotic supplements are classified as high levels.

### 2.4 Definition of covariates

Data on covariates were obtained from demographic, dietary, laboratory, and questionnaire sources, all of which are available to the public. Potential confounding factors include gender, age, ethnicity (Mexican American, Other Hispanic, Non-Hispanic Black, Non-Hispanic White, Other Races), household poverty-income ratio (high income, medium income and low income), smoking status (former smoker, current smoker, never smoker), alcohol consumption status (any alcohol use, no alcohol use), education level (more than high school, high school, less than high school), marital status (Living alone or Married/living with a partner), BMI, hypertension, and diabetes. Based on the poverty income ratio (PIR) published by the US government, family income was classified as low (PIR ≤ 1.3), medium (PIR >1.3–3.5), and high (PIR >3.5) (19). Diabetes was defined as self-reported and physician-diagnosed diabetes using insulin or oral hypoglycemic agents with a fasting blood glucose level of 7.0 mmol/L, oral glucose tolerance test 2 h post-meal blood glucose level of 11.1 mmol/L, random blood glucose level of 11.1 mmol/L, or glycosylated hemoglobin (HbA1c) level of 6.5% (20). Hypertension was defined as self-reported hypertension with a mean systolic blood pressure of 140 mm Hg or/and a mean diastolic blood pressure of 90 mm Hg on three consecutive measurements, or the use of hypotensive drugs (21).

### 2.5 Statistical analysis

The complex sampling design of NHANES was fully considered in the analysis, MEC sample weights were used, and sample weights were calculated according to the NHANES analysis guidelines. Continuous variables were described as weighted means (standard error, SE), and categorical variables were reported as weighted percentages and frequencies. The odds ratio (OR) and 95% confidence interval (CI) between dietary live microbial intake and severe headache or migraine were calculated using multivariate logistic regression analysis. Similar to a previous study, the analysis covered three models: crude model was not adjusted for variables, model 1 was adjusted for gender and age, and model 2 was adjusted for age, gender, PIR, race, education levels, marital status, smoke status, alcohol status, hypertension, diabetes, BMI (22). In addition, subgroup analyses were performed to examine heterogeneity among different subgroups by adding interacting terms and stratifying variables by age, gender, education levels, BMI and PIR. All statistical analyses used two-side *P*-values < 0.05 as the statistical significance criterion.

## 3 Results

### 3.1 Characteristics of study participants

Table 1 presents the basic characteristics of the 13,443 participants. The participants were classified according to the level of dietary live bacteria intake. In comparison with the low group, the subjects in the medium-high group were older, female, non-Hispanic White, had higher income, more educated, were of normal weight, were non-smokers, current drinkers, did not have high blood pressure, were not living alone, and did not have severe headache or migraine (all *P* < 0.05).

**Table 1:** Baseline characteristics of the study population by various dietary live microbes.

**Characteristic**	**Low *N* = 4,603 (33%)**	**Med *N* = 6,426 (46%)**	**High *N* = 2,414 (21%)**	***P*-value**
**Gender**				
Female	2,233 (48%)	3,437 (53%)	1,403 (56%)	<0.001
Male	2,370 (52%)	2,989 (47%)	1,011 (44%)	
Age	42 (31, 54)	45 (33, 59)	42 (31, 53)	<0.001
**PIR**				
High income	1,081 (34%)	1,945 (44%)	949 (52%)	<0.001
Low income	1,465 (28%)	1,583 (20%)	487 (15%)	
Medium income	1,637 (38%)	2,347 (37%)	797 (33%)	
**Race**				
Mexican American	849 (5.8%)	1,687 (8.7%)	492 (6.1%)	<0.001
Non-Hispanic Black	1,295 (16%)	1,026 (9.5%)	266 (5.9%)	
Non-Hispanic White	2,068 (66%)	3,299 (73%)	1,476 (79%)	
Other Hispanic	253 (7.1%)	258 (5.3%)	102 (5.0%)	
Other Races	138 (4.3%)	156 (3.4%)	78 (3.6%)	
**Education levels**				
<High school	818 (8.6%)	1,010 (6.6%)	262 (4%)	<0.001
High school	2,138 (47%)	2,513 (38%)	790 (31%)	
>High school	1,633 (45%)	2,894 (55%)	1,359 (65%)	
**Marital status**				
Living alone	1,703 (41%)	2,064 (34%)	787 (35%)	<0.001
Married/living with a partner	2,516 (59%)	3,995 (66%)	1,515 (65%)	
**Smoke status**				
Current	1,297 (33%)	1,179 (21%)	439 (20%)	<0.001
Former	1,097 (21%)	1,830 (27%)	672 (27%)	
Never	2,198 (46%)	3,414 (52%)	1,298 (53%)	
**Alcohol status**				
Any alcohol use	2,871 (70%)	4,091 (71%)	1,668 (76%)	0.001
No alcohol use	1,731 (30%)	2,335 (29%)	746 (24%)	
**Hypertension**				
Yes	1,540 (28%)	2,048 (28%)	666 (23%)	<0.001
No	3,063 (72%)	4,378 (72%)	1,748 (77%)	
**Diabetes**				
Yes	635 (9.5%)	879 (9.7%)	258 (7.3%)	0.020
No	3,968 (90.5%)	5,547 (90.3%)	2,156 (92.7%)	
**BMI group**				
<18.5	83 (2.4%)	77 (1.6%)	48 (2.5%)	<0.001
18.5–24.99	1,283 (32%)	1,895 (36%)	760 (37%)	
25–29.99	1,267 (29%)	1,904 (31%)	686 (29%)	
≥30	1,534 (36%)	1,970 (32%)	715 (31%)	
**Severe headache or migraine**				
Yes	1,073 (26%)	1,190 (20%)	488 (20%)	<0.001
No	3,530 (74%)	5,236 (80%)	1,926 (80%)	

BMI, body mass index; PIR, poverty income ratio.

Table 2 likewise describes the basic characteristics of the study population included. Two thousand seven hundred fifty-one exhibited severe headache or migraine among 13,443 participants. Compared to participants without severe headache or migraine, those who with severe headache or migraine were more likely to be female, younger, have lower income, lower levels of education, be current smokers, be obese, be smokers, and be non-drinkers. In addition, patients with severe headache or migraine consume less dietary live microorganisms (*P* < 0.001).

**Table 2:** Study population characteristics from NHANES 1999–2004 by severe headache or migraine.

**Characteristic**	**Control *N* = 10,692 (78%)**	**Severe headache or migraine *N* = 2,751 (22%)**	***P*-value**
**Gender**			
Female	5,206 (48%)	1,867 (67%)	**<0.001**
Male	5,486 (52%)	884 (33%)	
Age	45 (32, 58)	40 (30, 50)	**<0.001**
**PIR**			
High income	3,345 (45%)	630 (33%)	**<0.001**
Low income	2,617 (19%)	918 (30%)	
Medium income	3,820 (36%)	961 (37%)	
**Race**			
Mexican American	2,352 (7.0%)	676 (7.8%)	**0.002**
Non-Hispanic Black	2,006 (11%)	581 (12%)	
Non-Hispanic White	5,585 (73%)	1,258 (68%)	
Other Hispanic	452 (5.4%)	161 (7.6%)	
Other Races	297 (3.7%)	75 (3.9%)	
**Education levels**			
<High school	1,638 (6.4%)	452 (7.6%)	**<0.001**
>High school	4,807 (55%)	1,079 (48%)	
High school	4,227 (38%)	1,214 (44%)	
**Marital status**			
Living alone	3,593 (36%)	961 (38%)	0.3
Married/living with a partner	6,430 (64%)	1,596 (62%)	
**Smoke status**			
Current	2,174 (23%)	741 (31%)	**<0.001**
Former	3,022 (26%)	577 (20%)	
Never	5,482 (51%)	1,428 (49%)	
**Alcohol status**			
Any alcohol use	7,008 (73%)	1,622 (67%)	**<0.001**
No alcohol use	3,684 (27%)	1,129 (23%)	
**Hypertension**			
Yes	3,404 (26%)	850 (27%)	0.3
No	7,288 (74%)	1,901 (63%)	
**Diabetes**			
Yes	1,443 (9.4%)	329 (8.3%)	**0.012**
No	9,249 (90.6%)	2,422 (91.7%)	
**BMI group**			
<18.5	150 (1.8%)	58 (3.0%)	**<0.001**
18.5–24.99	3,172 (36%)	766 (32%)	
25–29.99	3,164 (31%)	693 (26%)	
≥30	3,219 (32%)	1,000 (39%)	
**Live microbes**			
Low	3,530 (32%)	1,073 (39%)	**<0.001**
Med	5,236 (47%)	1,190 (42%)	
High	1,926 (21%)	488 (19%)	

BMI, body mass index; PIR, the poverty income ratio. The meaning of the bold values indicates *p* < 0.05.

### 3.2 Association between live microbe intake and severe headache or migraine

**Figure 2:**
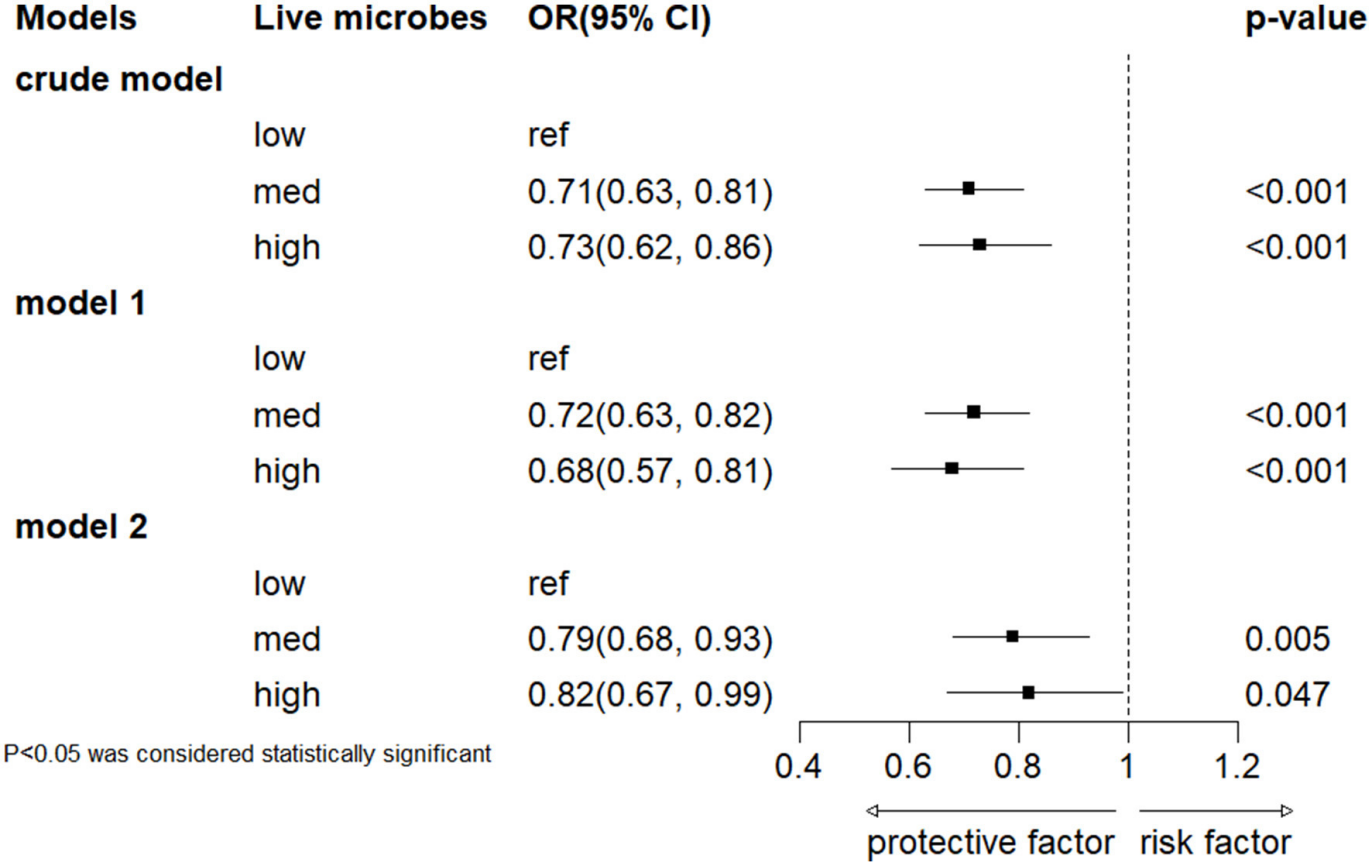
Association between dietary intake of live microbes and severe headache or migraine. Crude model included no covariates. Model 1 was adjusted for age and gender, and model 2 was adjusted for gender, age, PIR, race, education levels, marital status, smoke status, alcohol status, hypertension, diabetes and BMI.

The results of multiple regression analysis are shown in Figure 2. Overall, in all models, a higher intake of dietary live microorganisms is associated with a lower incidence risk of severe headache or migraine. Compared with the low dietary live microorganism group, the severe headache or migraine OR (95% CI) of the medium-high dietary live microorganism group is 0.71 (0.63–0.81) and 0.73 (0.62–0.86), respectively, in the unadjusted model. In model 1, adjusted according to age and gender, compared with the low dietary live microorganism group, the adjusted severe headache or migraine OR (95% CI) of the medium-high dietary live microbes group is 0.72 (0.63–0.82) and 0.68 (0.57–0.81). In model 2, after adjusting for age, gender, PIR, race, education levels, marital status, smoke status, alcohol status, hypertension, diabetes and BMI, compared with the low dietary live microbes group, the adjusted OR (95% CI) for severe headache or migraine in the medium-high dietary live microbes group is 0.79 (0.68–0.93) and 0.82 (0.67–0.99).

### 3.3 Subgroup analyses

**Figure 3:**
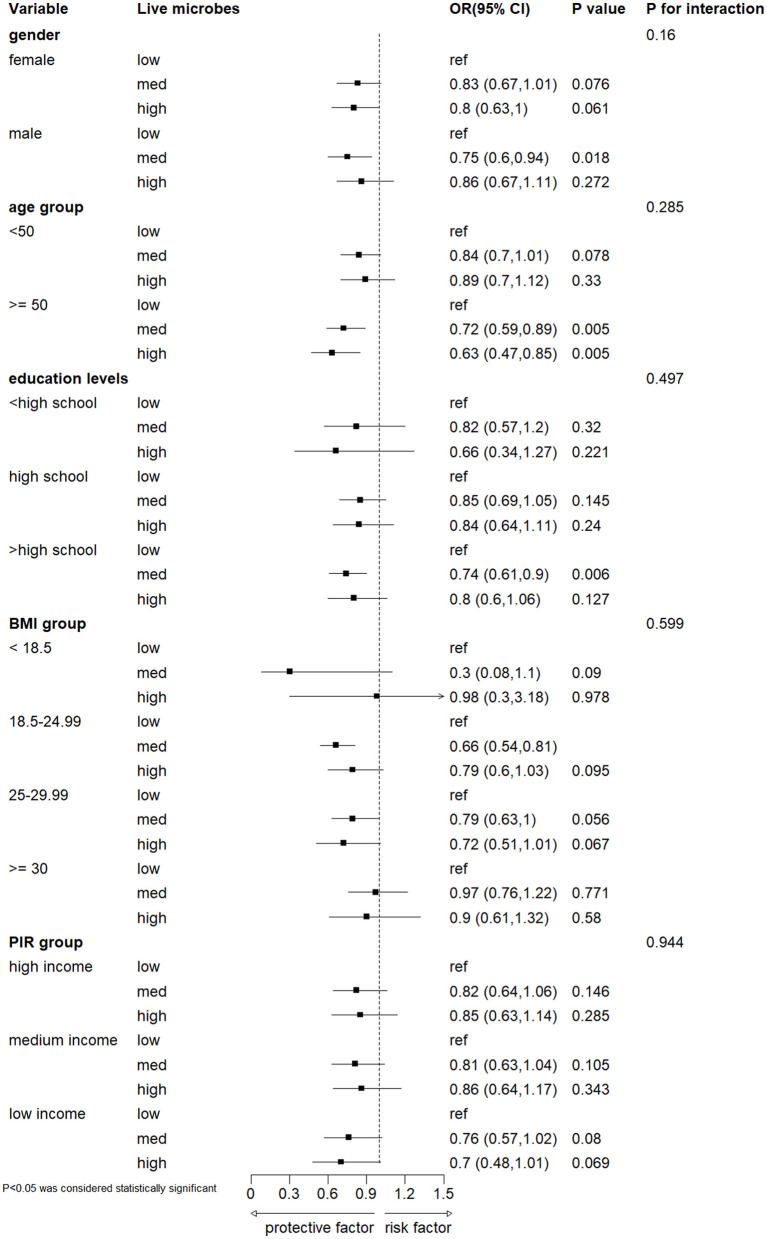
Subgroup analyses on the association between different dietary intake of live microbes and severe headache or migraine. Model was adjusted for gender, age, PIR, race, education levels, marital status, smoke status, alcohol status, hypertension, diabetes and BMI. BMI, body mass index; PIR, the poverty income ratio.

Subgroups were analyzed according to gender, age, education level, body mass index (BMI), and PIR grouping (Figure 3). The results showed no significant interaction between dietary active microbial intake and these stratified variables in terms of severe headache or migraine risk (interaction *P*-value > 0.05). Specifically, individuals aged ≥50 years had a decreased risk of developing severe headache or migraine in the moderate and high dietary active microbial intake groups. Male participants and those with a BMI of 18.5–24.99 had a reduced risk of developing severe headache or migraine in the moderate dietary active microbial intake group.

## 4 Discussion

In this study, we found that moderate-high dietary live microbes were negatively associated with the prevalence of severe headache and migraine. Probiotics were defined as “live microbes that, when administered in adequate amounts, confer a health benefit on the host” in 2001 by the Food and Agriculture Organization of the United Nations (FAO)/World Health Organization (WHO) (23). Bacteria genera such as bifidobacteria, *Escherichia coli*, lactic acid bacteria, and *Saccharomyces* are known probiotics (24). Human diet comprises a diverse array of products that serve as sources of probiotic strains. The quantity and diversity of microorganisms, including bacteria, yeasts, and molds, are contingent upon the type, origin, and degree of processing of the food. We can consider the gastrointestinal tract as an open ecosystem where microorganisms ingested through diet can regulate the composition and activity of resident microbial communities. Gut microbiota exhibits a rapid response to altered diets, potentially contributing to the diversity of dietary lifestyles in humans (25, 26).

The underlying mechanisms for the association between high dietary live microbe intake and severe headaches or migraine may be as follows. First, the microbiome-gut-brain axis theory has proposed a new possibility for the pathogenesis of migraine. Intestinal flora can participate in the bidirectional regulation of the gut and central nervous system through various ways, and then affect the host brain function. Simultaneously, the brain also modulates the motility and functionality of the gastrointestinal tract via hormonal mediators (27, 28). The dysfunction of the gut-brain axis has been linked to many neurological diseases, such as mood and anxiety disorders, multiple sclerosis, Parkinson disease, Alzheimer disease, and migraine (27–29). Second, in the gastrointestinal tract, immune cells and inflammatory mediators, such as interleukin IL-6, IL-1β, IL-18, and tumor necrosis factor alpha (TNF-α), interferon gamma (IFN-γ), have been identified as sensitizers of afferent nerve endings and are recognized as inducers of visceral pain (30). Some pro-inflammatory cytokines are associated with migraines and their levels increase during migraine attacks (31). A study has shown that commensal microbiota is fundamental for the development of inflammatory pain and the interactions between the symbiotic microbiome and the host play an important role in promoting adaptation to environmental stress, which can result in tissue damage, inflammation, and heightened pain perception (32). Third, the central nervous system (CNS) can modulate gut microbiota through both the sympathetic and parasympathetic systems, as well as by releasing neuroendocrine peptides. CGRP, a 37-amino acid peptide, is a product of alternative splicing of RNA from the calcitonin gene, plays a key role in the pathogenesis of migraine, and its role in the trigeminal vascular system is a new direction for the treatment and prevention of migraine (33). It is widely distributed in the peripheral and central nervous system (CNS) (34, 35). The CGRP signaling could be influenced by microbiota (36). CGRP, vasoactive intestinal peptide (VIP), Substance P (SP), and neuropeptide Y (NPY) are thought to have antibacterial effects on various strains of intestinal bacteria, such as Escherichia coli, Enterococcus faecalis, and Lactobacillus acidophilus, and are therefore presumed to be involved in the bidirection relationship between the gut and the brain (36). Additionally, Probiotic supplements may regulate migraine attacks, but the underlying mechanism of action is not fully understood. Studies have shown that changes in the composition of the gut microbiota and related metabolites, such as short-chain fatty acids (SCFAs), and the release of cytokines, such as TNF-α, can modulate migraine-like pain in animal models (37). Probiotics impact migraine by altering the composition of the gut microbiota, reducing intestinal permeability, and decreasing the transfer of these pro-inflammatory substances (38, 39).

A randomized double-blind controlled trial evaluated the effects of taking 14 probiotic mixtures or a placebo daily for 8 weeks in patients with chronic migraine and 10 weeks in patients with episodic migraine. The results suggested that the use of probiotics significantly improved the frequency and severity of migraine and reduced the use of abortion medications (40). In an open-label trial of 40 migraine patients, supplementation with probiotics + minerals + vitamins + herbs for 12 weeks resulted in significant improvements in quality of life for about 80% of the subjects and pain relief for more than half of the migraine patients. In further studies, a probiotic mixture supplemented with seven bacterial strains reduced migraine attack frequency by about a quarter and also reduced migraine-related disability (41, 42). It is important to note that current research results are inconsistent, with some studies showing a positive effect of probiotics on migraines, while others have found no significant effect (39).

Based on the research findings presented in this article, consuming foods that supply dietary live microbes can offer benefits to adults in preventing migraine attacks. Ingesting live microbes through diet is generally considered safer than pharmacological interventions and has fewer adverse effects (43). However, probiotics may not be suitable for all individuals and are contraindicated in certain patient populations, including those with cancer, autoimmune diseases, transplant recipients, and the elderly. Those who with compromised immune systems and severe illnesses should be cautious when considering probiotics.

To our knowledge, this is the first study to investigate the relationship between dietary live microbes and the prevalence of severe headache and migraine in a large, representative US population. The data utilized in this study were sourced from the National Health and Nutrition Examination Survey (NHANES), a nationally representative database in the United States, and underwent rigorous quality control measures to ensure their validity. Nevertheless, the current study also has several limitations. First of all, it's worth noting that the study used a cross-sectional design, that may limit its ability to establish a cause-and-effect connection between dietary intake of live microbes and migraine occurrence. Secondly, the classification methods used in our study to determine the content of live microorganisms in feed were developed by leading experts in various disciplines through collaborative discussions and an extensive literature review. The approach has significant advantages in terms of efficiency and applicability. However, participants were initially divided into three groups, the low dietary live microbe group, the medium dietary live microbe group and the high dietary live microbe group. The simple classification without accurate calculation is easy to cause errors. The accurate detection and calculation of live microorganisms in daily diet remains to be further explored. Thirdly, even after accounting for as many confounding variables as possible, other confounding factors may still affect the results. Fourthly, 24-h food recall relies on participants' memory and self-reports, which can lead to overestimation or underestimation of food intake. The participants could not be able to recall all foods and beverages consumed accurately in a day, especially for those consumed less frequently. Finally, the sample in our study was drawn from the U.S. population, so it's necessary to validate the results in other countries.

## 5 Conclusion

In conclusion, current evidence suggests that living Microbe Intake may have positive health effects for migraine patients by improving the gut microbiome. Future research may reveal new therapeutic targets, leading to more treatment options for migraine sufferers.

## Data Availability

Publicly available datasets were analyzed in this study. This data can be found at: https://www.cdc.gov/nchs/nhanes/index.htm.
